# Dupilumab‐Induced Erythrodermic Psoriasiform Dermatitis in Chronic Actinic Dermatitis: Case Report and Innovative Treatment Approach

**DOI:** 10.1002/ccr3.9590

**Published:** 2024-11-22

**Authors:** Joe Khodeir, Maya Habre

**Affiliations:** ^1^ Department of Dermatology, Faculty of Medicine and Medical Sciences, Saint Georges Hospital University Medical Center University of Balamand Balamand Lebanon; ^2^ Division of Dermatology, Saint Georges Hospital University Medical Center Saint George University of Beirut Beirut Lebanon

**Keywords:** chronic actinic dermatitis, dupilumab, dupilumab‐induced psoriasis, erythrodermic psoriasis, psoriasiform dermatitis

## Abstract

The off‐label use of dupilumab has proven to be an effective treatment option for severe chronic actinic dermatitis (CAD). We present the first case of CAD presenting with dupilumab‐induced erythrodermic psoriasiform dermatitis. A modified, spaced dupilumab dosing regimen, combined with cyclosporine, successfully managed both conditions, offering a promising strategy for managing dupilumab adverse effects while maintaining control over chronic actinic dermatitis.

## 1 Introduction

Chronic actinic dermatitis (CAD) is a rare, photosensitive condition characterized by eczematous, lichenified plaques in sun‐exposed areas [1]. Treatment often involves sun protection, steroids, and immunosuppressants, but many patients experience limited success, impacting their quality of life [1].

Dupilumab, an IL‐4 receptor alpha antagonist, is effective for various inflammatory skin conditions but has been associated with adverse effects, including the induction of psoriasis. Erythrodermic psoriasis is a severe, life‐threatening form that requires prompt diagnosis and treatment [2].

We present a 72‐year‐old male with a long‐standing history of biopsy‐proven CAD who developed erythrodermic psoriasiform dermatitis following dupilumab treatment. This case highlights the importance of recognizing drug‐induced complications and suggests a possible association between CAD and psoriasis. Additionally, we discuss a novel approach to managing both conditions by switching to cyclosporine, then reintroducing dupilumab with a modified dosing regimen, which successfully controlled both CAD and psoriasiform dermatitis without recurrence of the adverse effects.

## 2 Case History/Examination

**FIGURE 1:**
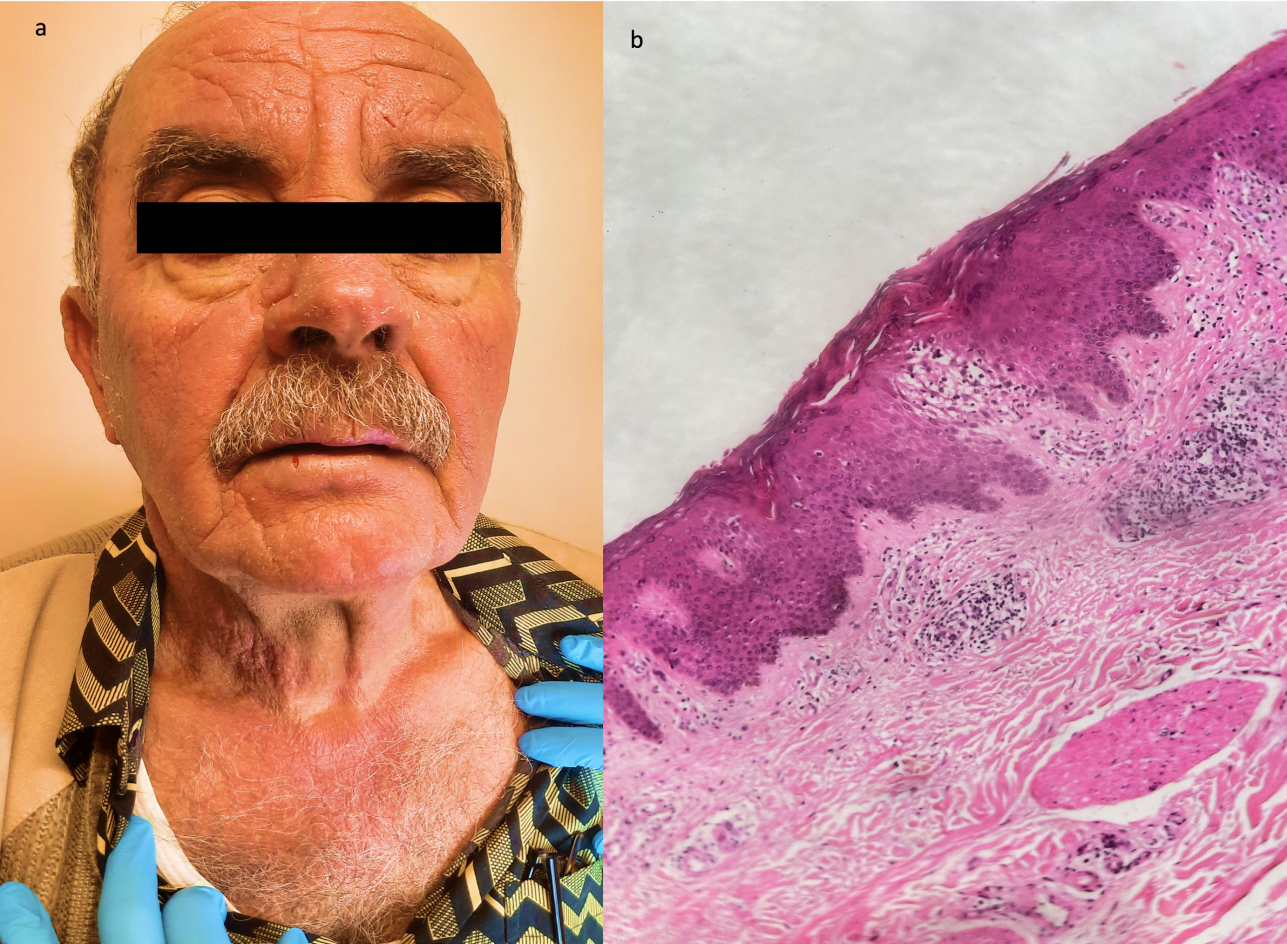
(a, b) Hyperkeratotic, lichenified and erythematous plaques seen on photodistributed areas only (a—face, neck and b—hands).

A 72‐year‐old male presented with a 12‐year history of biopsy‐proven CAD, manifesting as eczematous, lichenified plaques in photodistributed areas (Figure 1a,b). Previous treatments, including topical and systemic steroids, oral hydroxychloroquine, methotrexate, and strict sun protection, were ineffective, leading to severe disease progression. A short course of cyclosporine at 300 mg/day led to improvement; however, the patient's condition worsened when the dose was reduced to 100 mg.

## 3 Methods (Differential Diagnosis, Investigations and Treatment)

**FIGURE 2:**
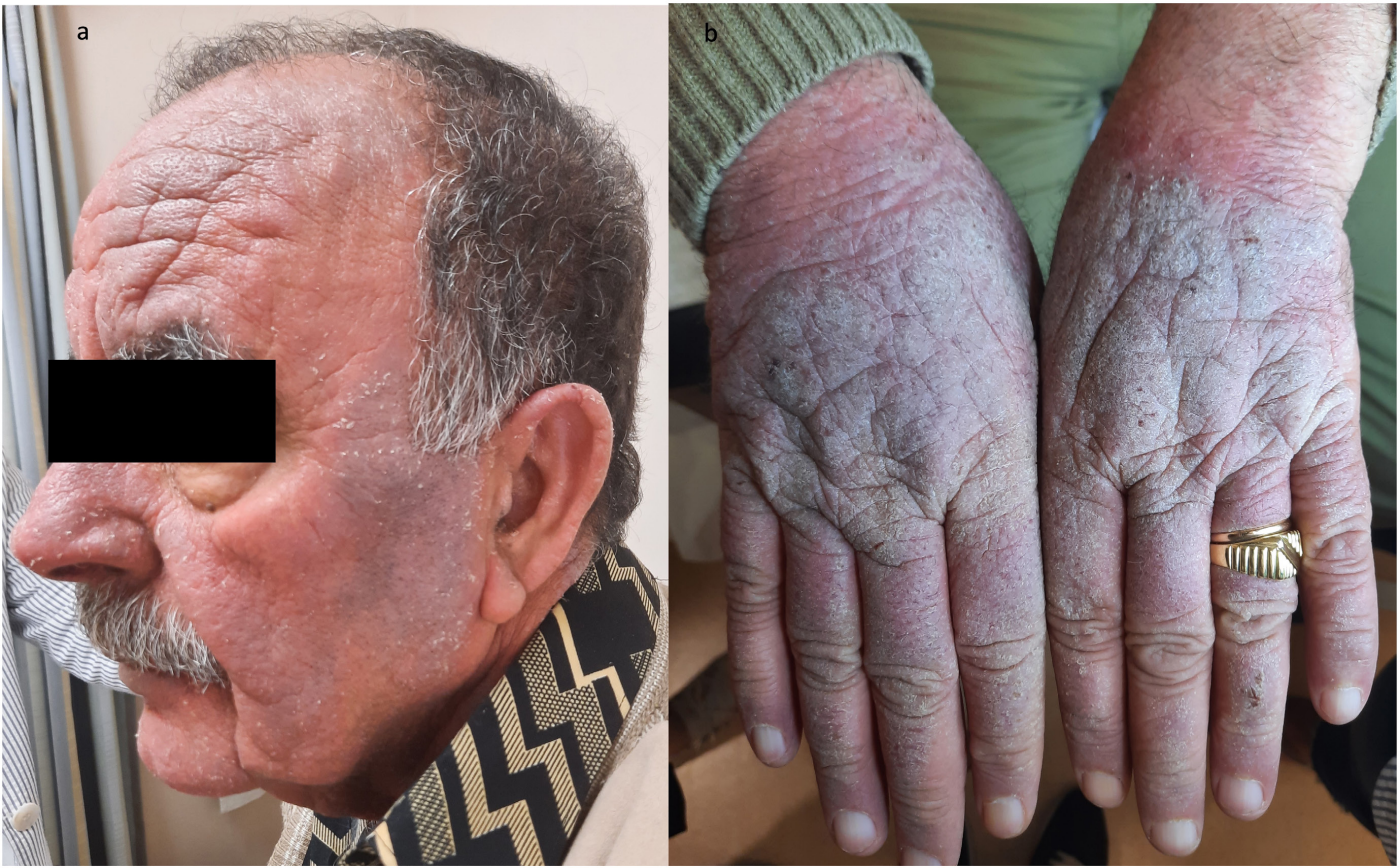
(a) severe erythroderma is seen after dupilumab therapy and (b) HE ×100 stain showing hyperkeratosis, acanthosis, parakeratosis, and elongation of the rete ridges compatible with a psoriasiform dermatitis.

Following a severe flare, another biopsy ruled out mycosis fungoides and confirmed severe CAD. Given the recalcitrant nature of his condition, dupilumab was initiated with a loading dose of 600 mg followed by 300 mg every 2 weeks. The patient exhibited significant improvement within 2 months, achieving near‐complete remission. It is worth noting that pruritus subsided as early as 4 weeks into therapy. However, after 3 months of therapy, new hyperkeratotic erythematous plaques appeared on his body, combined with severe generalized erythroderma (Figure 2a).

An urgent biopsy was performed to rule out erythrodermic chronic actinic dermatitis, also known as actinic reticuloid, versus dupilumab‐induced erythrodermic psoriasis. Histopathology revealed features of psoriasiform dermatitis, including hyperkeratosis, acanthosis, parakeratosis, elongation of the rete ridges and psoriasiform hyperplasia (Figure 2b). Based on these findings, a diagnosis of dupilumab‐induced erythrodermic psoriasis was made. Dupilumab was discontinued, and oral cyclosporine (300 mg daily) was started, leading to significant clinical improvement of both erythroderma and CAD.

## 4 Conclusions and Results (Outcome and Follow‐Up)

At follow‐up 3 months later, the patient showed no relapse of either condition. A decision was made to re‐start the patient on dupilumab without a loading dose, administering a maintenance dose of 300 mg monthly, while simultaneously reducing the dose of cyclosporine to 100 mg daily. Three months after this adjusted regimen, there was no recurrence of erythrodermic psoriasiform dermatitis, nor a relapse of CAD. The patient has been successfully maintained on this regimen, demonstrating that a modified dupilumab dosing schedule, combined with a lower dose of cyclosporine, can be an effective strategy for managing both conditions without triggering adverse effects.

## 5 Discussion

Off‐label use of dupilumab has shown efficacy in treating CAD, with case series and reports documenting significant improvement in patients unresponsive to conventional therapies [1]. In all reported cases, the dosing regimen of dupilumab followed the standard protocol used for atopic dermatitis, starting with a 600 mg loading dose, followed by 300 mg biweekly. This approach consistently led to significant improvement without any reported major adverse effects. In some cases, dupilumab was combined with hydroxychloroquine, sun protection, and topical steroids to enhance treatment outcomes [1, 3, 4]. Nevertheless, dupilumab can induce various types of psoriasiform dermatitis. According to a review by Su and Zeng [2], among patients with dupilumab‐associated psoriasiform dermatitis, the reported lesion types were plaque psoriasis, pustular psoriasis, guttate psoriasis, erythrodermic psoriasis, reverse psoriasis, and sebopsoriasis.

In our case, erythrodermic psoriasis occurred, which is considered a dermatologic emergency requiring rapid diagnosis and treatment to reduce mortality [5]. In the literature, there are only two reported cases of erythrodermic psoriasis induced by dupilumab. Both cases were postulated to have been misdiagnosed as atopic dermatitis, for which dupilumab was initiated, subsequently transforming undiagnosed psoriasis into erythrodermic psoriasis [6, 7].

In our patient, however, the diagnosis of CAD was confirmed by multiple biopsies and the characteristic photosensitive rash, thus an undiagnosed psoriasis was not the case. Therefore, the only explanation could be the possible association between CAD and psoriasis. A potential hypothesis for why an erythrodermic psoriasis appeared in our case could be related to the association between psoriasis and CAD. A case reported by Sahoo and Kumar [8] described a patient with psoriasis vulgaris who developed lesions of CAD, suggesting a possible relationship between the two conditions. Another case by Fujii et al. [9] described the development of CAD during phototherapy for psoriasis. These instances indicate that there might be an underlying connection between CAD and psoriasis, which could explain the occurrence of erythrodermic psoriasis in our patient following the use of dupilumab.

The role of dupilumab in this context is crucial. It shifts the immune response from a Th2‐dominant to a Th1‐dominant profile [10]. In the setting of CAD, which is characterized by Th2‐driven inflammation [1], this shift could unmask or exacerbate Th1‐driven pathologies, such as psoriasis [10]. As a result, erythrodermic psoriasis may emerge as a manifestation of this immune shift, particularly in patients with a predisposition to both conditions.

In challenging cases like ours, long‐term use of cyclosporine is not feasible due to its potential adverse effects, especially in elderly patients. Discontinuation of cyclosporine often leads to a flare of CAD, as seen in our patient [11, 12, 13]. Given the proven efficacy of dupilumab in managing his condition, we decided to reintroduce dupilumab at a modified dosing regimen, 300 mg monthly without a loading dose, while continuing cyclosporine at a reduced dose of 100 mg daily. This decision was made with careful consideration and close patient monitoring to promptly identify any recurrence of psoriasiform dermatitis.

Remarkably, at this adjusted dosage regimen, the patient achieved clearance of both CAD and psoriasiform dermatitis without experiencing further adverse effects. This approach suggests that a spaced dosing regimen of dupilumab may effectively manage CAD while minimizing the risk of psoriasiform dermatitis.

Our long‐term goal is to discontinue cyclosporine and maintain the patient solely on dupilumab, which, if successful, would reduce the risk of cyclosporine‐related adverse effects and provide a sustainable treatment option. This novel dosing strategy could represent a promising alternative for reducing dupilumab's adverse events, particularly psoriasiform dermatitis, in patients with complex treatment needs.

## 6 Conclusion

This case underscores the importance of vigilance for adverse effects when treating CAD with dupilumab, particularly the potential for inducing erythrodermic psoriasis, a dermatologic emergency. While previous reports suggest misdiagnosis as atopic dermatitis, our patient's multiple biopsy‐confirmed diagnosis of CAD and photosensitive rash point to a different pathogenesis. The possible association between CAD and psoriasis, as observed in other cases, may account for this rare complication. Clinicians should consider this potential relationship and monitor patients closely, ensuring prompt intervention to reduce morbidity and mortality.

Our experience suggests that a spaced dosing regimen of dupilumab might be a viable strategy to mitigate adverse events, such as psoriasiform dermatitis, while still effectively managing CAD. This approach allowed for the successful management of both CAD and erythrodermic psoriasis in our patient. Our long‐term goal is to discontinue cyclosporine and maintain the patient solely on dupilumab, which would minimize the risk of cyclosporine‐related adverse effects. Further research is needed to elucidate the mechanisms by which dupilumab can induce erythrodermic psoriasis and to refine dosing strategies to optimize patient outcomes and minimize adverse events.

## Author Contributions

**Joe Khodeir:** investigation, writing – original draft, writing – review and editing. **Maya Habre:** conceptualization, investigation, supervision, validation, writing – review and editing.

## Ethics Statement

Written informed consent was taken from the patient to publish the figures.

## Conflicts of Interest

The authors declare no conflicts of interest.

## Data Availability

The data used to support the findings of this study are included within the article.
